# Supplementary material for: FSTL1 Suppresses Triple-Negative Breast Cancer Lung Metastasis by Inhibiting M2-like Tumor-Associated Macrophage Recruitment toward the Lungs
Source: Diagnostics (Basel). 2023 May 12;13(10):1724. doi: 10.3390/diagnostics13101724 (PMC10217361; doi:10.3390/diagnostics13101724)
Supplement: Supplementary file 1 [file diagnostics-13-01724-s001.zip › diagnostics-2333489-supplementary.pdf]

FSTL1 suppresses triple-negative breast cancer lung metastasis by inhibiting M2-like tumor-associated macrophage recruitment toward the lung

Supplementary materials

Figure-S1 The Function of TAMs

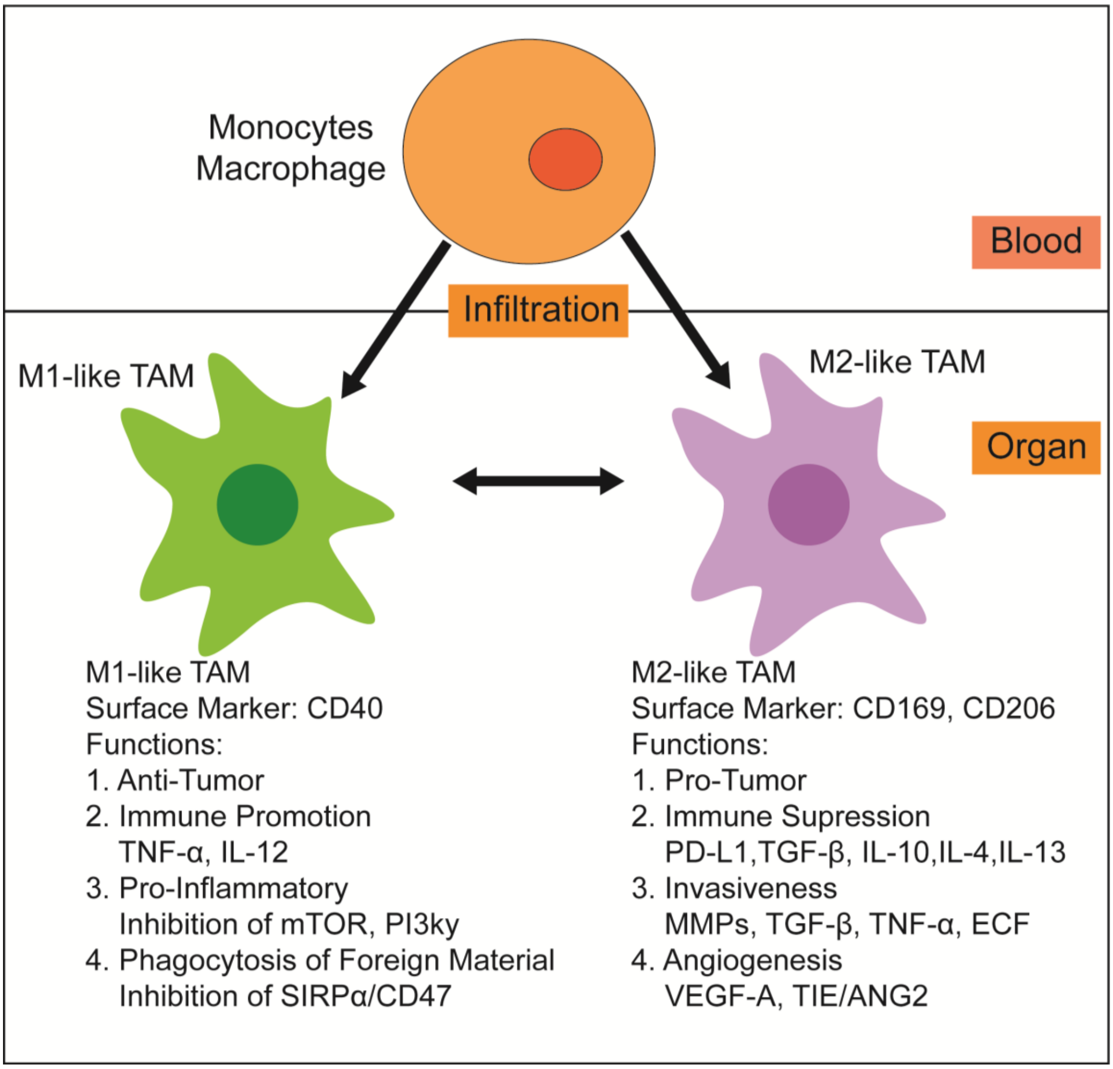

1. Christofides A, Strauss L, Yeo A, Cao C, Charest A, Boussiotis VA. The complex role of tumor-infiltrating macrophages. Nat Immunol. 2022 Aug;23(8):1148-1156.

2. Ngambenjawong C, Gustafson HH, Pun SH. Progress in tumor-associated macrophage (TAM)-targeted therapeutics. Adv Drug Deliv Rev. 2017 May 15;114:206-221

Figure-S2 The function of FSTL1

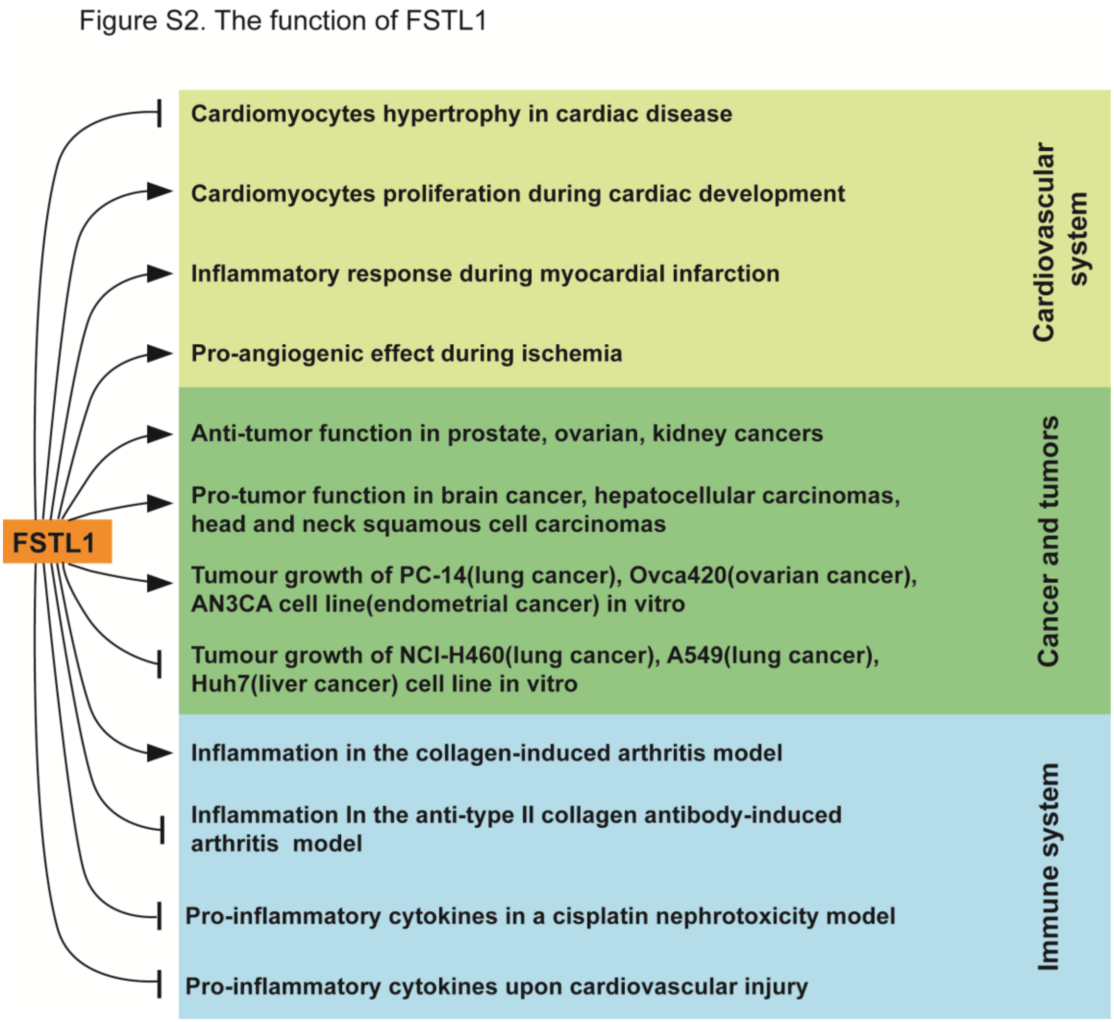

1. Mattiotti A, Prakash S, Barnett P, van den Hoff MJB. Follistatin-like 1 in development and human diseases. Cell Mol Life Sci. 2018 Jul;75(13):2339-2354.

2. Li W, Alahdal M, Deng Z, Liu J, Zhao Z, Cheng X, Chen X, Li J, Yin J, Li Y, Wang G, Wang D, Tang K, Zhang J. Molecular functions of FSTL1 in the osteoarthritis. Int Immunopharmacol. 2020 Jun;83:106465.

Table S1 Main result of the present study.

1	FSTL1 mRNA expression decreases in human breast cancer and its various subtypes
2	Breast cancer patients with high FSTL1 expression show prolonged survival
3	FSTL1 can not promote the proliferation of TNBC in situ but remarkably increase its lung metastasis
4	Fstl1+/- mice exhibit increased M2 macrophages deposition to the lung
5	FSTL1 inhibited M2-like TAMs migration toward 4T1 triple-negative breast cancer cells
6	FSTL1 inhibited the secretion of CSF1, VEGF-α, and TGF-β in 4T1 triple-negative breast cancer cells
